# Supplementary material for: Splice-Junction-Based Mapping of Alternative Isoforms in the Human Proteome
Source: Cell Rep. Author manuscript; Available in PMC 2020 Jan 15. (PMC6961840; doi:10.1016/j.celrep.2019.11.026)
Supplement: 3 [file NIHMS1546469-supplement-3.zip › DF2/PXD000561/Heart-28-Q99959-QITGLLWNLSSNDK.pdf]

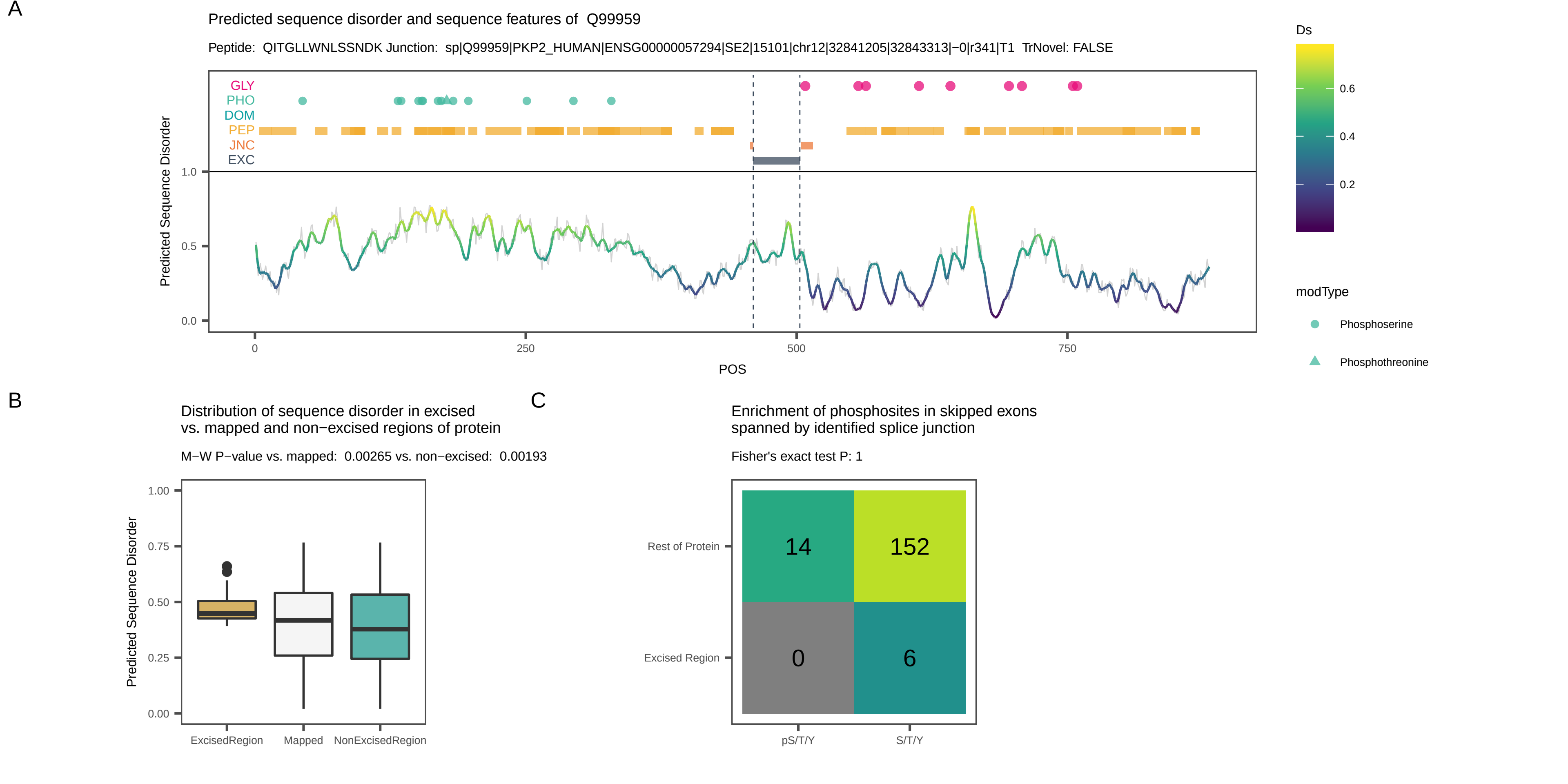
A
Predicted sequence disorder and sequence features of Q99959
Peptide: QITGLLWNLSSNDK Junction: sp|Q99959|PKP2_HUMAN|ENSG00000057294|SE2|15101|chr12|32841205|32843313|−0|r341|T1 TrNovel: FALSE
GLY
PHO
DOM
PEP
JNC
EXC
Predicted Sequence Disorder
1.0
0.5
0.0
0
250
500
750
POS
Ds
0.6
0.4
0.2
modType
Phosphoserine
Phosphothreonine
B
Distribution of sequence disorder in excised vs. mapped and non−excised regions of protein
M−W P−value vs. mapped: 0.00265 vs. non−excised: 0.00193
Predicted Sequence Disorder
1.00
0.75
0.50
0.25
0.00
ExcisedRegion
Mapped
NonExcisedRegion
C
Enrichment of phosphosites in skipped exons spanned by identified splice junction
Fisher's exact test P: 1
Rest of Protein
Excised Region
14
152
0
6
pS/T/Y
S/T/Y
